# Kinetic Walking Energy Harvester Design for a Wearable Bowden Cable-Actuated Exoskeleton Robot

**DOI:** 10.3390/mi13040571

**Published:** 2022-04-03

**Authors:** Yunde Shi, Mingqiu Guo, Heran Zhong, Xiaoqiang Ji, Dan Xia, Xiang Luo, Yuan Yang

**Affiliations:** 1 Department of Mechanical Engineering, Southeast University, Nanjing 210096, China; ydshi@seu.edu.cn (Y.S.); gmq337@seu.edu.cn (M.G.); dxia@seu.edu.cn (D.X.); luox@seu.edu.cn (X.L.); 2 Department of General Mechanics and Fundamentals of Mechanics, College of Engineering, Peking University, Beijing 100871, China; heranzhong@stu.pku.edu.cn; 3 Shenzhen Institute of Artificial Intelligence and Robotics for Society, The Chinese University of Hongkong, Shenzhen 518172, China; jixiaoqiang@cuhk.edu.cn; 4 Key Laboratory of Micro-Inertial Instrument and Advanced Navigation Technology, Ministry of Education, School of Instrument Science and Engineering, Southeast University, Nanjing 210096, China

**Keywords:** bowden cable actuation, energy harvesting, electromagnetic generator, soft exoskeleton robot, biomechanics

## Abstract

Over the past few decades, wearable exoskeletons of various forms have been developed to assist human activities or for rehabilitation of movement disorders. However, sustainable exoskeletons with efficient energy harvesting devices still have not been fully explored. In this paper, we propose the design of a lightweight wearable Bowden-cable-actuated soft exoskeleton robot with energy harvesting capability. Unlike previous wearable exoskeletons, the presented exoskeleton uses an electromagnetic generator to both harvest biomechanical energy and to output mechanical torque by controlling an operation mode relay switch based on a human’s gait. Moreover, the energy-harvesting module also acts as a knee impact absorber for the human, where the effective damping level can be modulated in a controlled manner. The harvested energy is regulated and stored in super capacitors for powering wireless sensory devices when needed. The experimental results show an average of a 7.91% reduction in thigh muscle activity, with a maximum of 3.2 W of electric power being generated during movement downstairs. The proposed design offers important prospects for the realization of lightweight wearable exoskeletons with improved efficiency and long-term sustainability.

## 1. Introduction

Wearable exoskeletons are external supporting structures for assisting human activities. Thus far, numerous exoskeletons have been developed and put to use in a variety of fields with diverse functionalities. The lower extremity exoskeleton robot (BLEEX), designed by the research group at the University of California, Berkeley, was able to assist infantrymen for heavy load carrying [1]. The RoboKnee exoskeleton, designed by Pratt et al., could provide assistive torque to the knee joint for enhancing strength and endurance during human walking [2], where the user’s intent was determined through the knee joint angle and ground reaction forces. A soft, wearable robotic device was developed by Park et al. for active knee motions using flat pneumatic muscles [3]. Their elastomer muscles demonstrated an initial contraction force of 38N and maximum contraction of 18 mm with 104 kPa of input pressure. Malcolm et al.’s exoskeleton was capable of assisting plantarflexion and reducing the human metabolic cost by means of controlling the pneumatic actuation time [4]. A metabolic cost reduction of 0.18 ± 0.06 W/kg or 6 ± 2% (standard error of the mean) below the cost of walking without an exoskeleton was reported if actuation started just before opposite leg heel contact. Mooney et al.’s autonomous exoskeleton provides the ankle joint with positive torque as the foot pushes off the ground, with a metabolic cost reduction of approximately 35 W being observed in the experiment compared with normal walking conditions without wearing the exoskeleton [5]. The powered ankle-foot orthoses developed by Norris et al. both increased human walking speeds and reduced the metabolic costs by providing augmented plantarflexion power [6]. In the study, eight out of nine young adults increased their preferred walking speeds when the push-off power was augmented (1.18 ± 0.16 to 1.25 ± 0.16 m/s, *p* = 0.03), while a similar but non-significant trend in the preferred walking speed was observed for the older adults. Soft exoskeletons have also been developed in recent years for better user comfort and wearing synergy in light of the traditional rigid and stiff exoskeleton designs [7].

Most of the abovementioned exoskeletons, however, require external power sources to operate. These powered exoskeletons may face energy problems for prolonged missions where an energy resupply is unavailable, especially under heavy load conditions. The bulky exoskeletons, such as HAL and BLEEX, have quite limited operation hours and could simply turn into an impediment or burden to the wearer once their energy sources run out. Technically, the duration of the exoskeleton could be made longer by adding more energy resources(e.g., more fuel or larger batteries with higher capacities). The additional weight added makes it impractical for the user to travel long distances carrying the heavy exoskeleton. Therefore, alternative solutions are needed for the design of lightweight and durable exoskeleton systems to deal with these energy issues [8,9].

One possible way to extend the operation times of exoskeletons is by extracting energy from the environment [10]. Environmental energies such as wind, sunlight, and temperature could be rich in overall quantity. However, they normally have a low energy density and may not be available in all situations [11] (e.g., little solar energy available during rainy days). These issues make it difficult to use environment energies for powering exoskeletons sustainably and reliably. Another possibility is to extract energy from human body heat using thermoelectric generators. In the study of Basset et al., a poly-SiGe-based 800 × 800 × 8000 μm μTEG was made to convert the temperature gradient formed between the body heat (310 K) and an ambient temperature of 298 K. The power generated was found to be 1.062 × 10^−6^ W for a single μTEG [12]. Other developments for various thermoelectric devices have also been reported. Recently, an energy-harvesting device using a thin layer of polydimethylsiloxane (PDMS) wrapped on the wrist was designed and tested. It was able to produce more than 10 µW/cm^2^ of power at room temperature when the human was walking at a normal speed [13]. While the energy produced with this method is of only microwatts, the output is sufficient for low-power consumption electronics, such as wireless electrocardiography. This approach, however, requires a large area of thermoelectric coverage on a human’s body skin for additional power output, causing significant discomfort when implemented.

The human body is a rich energy resource with the help of metabolism [14]. As a human is under motion, a large amount of kinetic energy is created by the movements of the limbs and body. Studies show that more than 100 W is consumed to move the lower limbs when a person of normal weight is walking at regular speeds [15]. Currently, foot strikes, body inertia, and vibration are the three major forms of human energy during walking. There have been reports on embedded insoles designed inside shoes [16,17] which harvest the foot–ground contact energy during walking [18]. The insoles are able to generate 250 mW of electricity during normal walking, powering a load radio [19]. However, the insole mechanism interferes with the user’s normal walking gait to some extent, especially when the insole’s output power is high [20]. The inertia energy associated with the motion of the human body’s center of mass is another important source. Various mechanisms (also known as “backpacks”) have been designed to convert the up and down movement to rotation, which subsequently drives generators to produce electric power [21]. These “backpacks”, however, are usually very bulky, adding extra burden to the human body during walking, and the aperiodic, low-frequency, and time-varying characteristics of inertia motion make energy harvesting rather difficult and less efficient for “backpacks” [22]. Over the years, some unpowered exoskeletons have also been proposed with energy harvesting abilities. A passive lower limb exoskeleton with artificial tendons was designed by Dijk et al. to reduce human joint load during walking [23], but the energy expenditure during walking with the exoskeleton is higher than normal walking. The unpowered knee exoskeleton designed by Xie et al. reduced the metabolic energy cost and generated 5.3 W during natural human walking at 4.5 km/h [24]. However, the proposed cable–pulley transmission system could not fully compensate for the angular variation around the hip joint when walking. Donelan et al. developed a knee brace which harvests the kinetic walking energy during deceleration of the lower limbs. The knee brace is able to output almost 5 W of electricity in the generative braking mode and 7 W in the continuous generation mode without adding too much extra burden to the user’s lower limbs [25]. However, the possible issue of knee brace misalignment was not considered in depth, as ideal mounting of the knee brace was assumed. Recently, triboelectric generators (TEGs) have attracted many researchers’ interest, where flexible structures have been designed and fabricated using nanotechnology to harvest human energy during exercise [26,27]. The designed TEGs, along with the biofuel cells (BFCs) by Lu et al., could provide sustainable power to a microwatt-rated wristwatch with a liquid crystal display (LCD) for more than 30 min, even after the human user stops moving [28]. The prolonged power delivery in this design, however, relies on the electroenzymatic reactions of sweat metabolites, which severely limits its generalized applications.

To harvest the kinetic energy of the human body, devices using other harvesting methods have also been explored and developed. Berdy et al. designed a magnetic levitation vibration energy harvester and studied its power output for participants with different body parameters (e.g., height) [29]. The reported power output was 71 μW (at 3 mph) and 342 μW (at 6 mph) when the human participants were running on a treadmill with the harvesting device. Additionally, to minimize the effects of damping, Berdy et al. studied the effect of the angle of attachment and damping reduction techniques using low-friction materials and a guide rail system, which improved power output by over 50% when compared with the suboptimal design. Magno et al. evaluated and integrated a highly efficient kinetic harvester circuit to power autonomous wearable devices. The designed Kinetron Micro Generator System 26.4 (MSG) was able to harvest up to 280 μJ from a single human movement and up to 1.1 J per day using a passive rectifier [30]. Their results were extremely promising for small wearable kinetic harvesting and demonstrated the instrumental application of this new generation of kinetic energy harvester in the design of many self-sustainable wearable devices. To collect the low-level kinetic energy present in all moving systems, Gljuscic et al. addressed the intrinsic problem of piezoelectric kinetic energy harvesting devices for wearable medical sensors [31]. In their study, complex numerical models comprising modal, harmonic, and transient analysis were created, and an optimized harvester geometry with an excitation mechanism was proposed to overcome the random nature of excitations generated by human motions. Specialized electrical circuitry was also designed and tested for efficient power management of their piezoelectric energy harvesting system.

Energy harvesting using non-traditional media has also been reported. Jia et al. designed a human kinetic energy harvesting device based on liquid metal magnetohydrodynamics [32]. Krupekin et al. used reverse electrowetting as a new approach to high-power energy harvesting, which increased the output power density to as high as 103 W/m^2^ [33]. Other studies involved energy harvesting using electrostatic generators. Such harvesters are inherently electrostatic capacitive energy converters, which can operate as vibration-to-electricity converters [34]. The major advantage of such an approach is the small size of the electrostatic generators, which is particularly useful for portable microelectromechanical systems when placed on the human body during walking [35]. Their efficiency, however, is usually low and requires a so-called “pre-charging” process to initiate energy conversion [36,37].

This paper proposes a flexible Bowdencable-actuated lower limb knee exoskeleton with the capability of both assisting human movements and harvesting negative work during the gait cycle. Instead of using separate devices for power assistance and energy harvesting, the exoskeleton uses a single electromagnetic unit which is controlled by an operation mode relay switch based on the user’s gait cycle. During lower limb acceleration or movement upstairs, the electromagnetic unit outputs power to the knee joint for assistance while it scavenges biomechanical energy during deceleration or movement down stairs. As the electromagnetic unit harvests the negative work, it also acts as a knee impact absorber. Unlike conventional mechanical dampers, the effective electrical damping of the impact absorber during the energy harvesting process can be adjusted and regulated by the microcontroller based on the user’s specific needs during operation. The harvested energy is regulated and stored in super capacitors for powering wireless electronics when needed. The experimental results show an average of a 7.91% reduction in thigh muscle activity, with a maximum of 3.2 W of electric power being generated during movement down stairs. The proposed design offers important prospects for the realization of lightweight wearable exoskeletons with improved efficiency and long-term sustainability.

The rest of the paper is organized as follows. Section 2 describes the knee exoskeleton design and energy flow of the system. Section 3 is about the modeling and simulation of the human-exoskeleton dynamics, the Bowdencable transmission system, power generation, and controllable knee joint damping. The mode switching criteria and associated circuit design for the harvesting, regulation, storage, and discharge of energy are presented in Section 4. The experimental results are provided in Section 5 with the analysis of EMG muscle activity and discussion on the harvested power, followed by our conclusions in Section 6.

## 2. Knee Exoskeleton Design and Energy Flow Analysis

### 2.1. Structural Design of the Knee Exoskeleton

**Figure 1:**
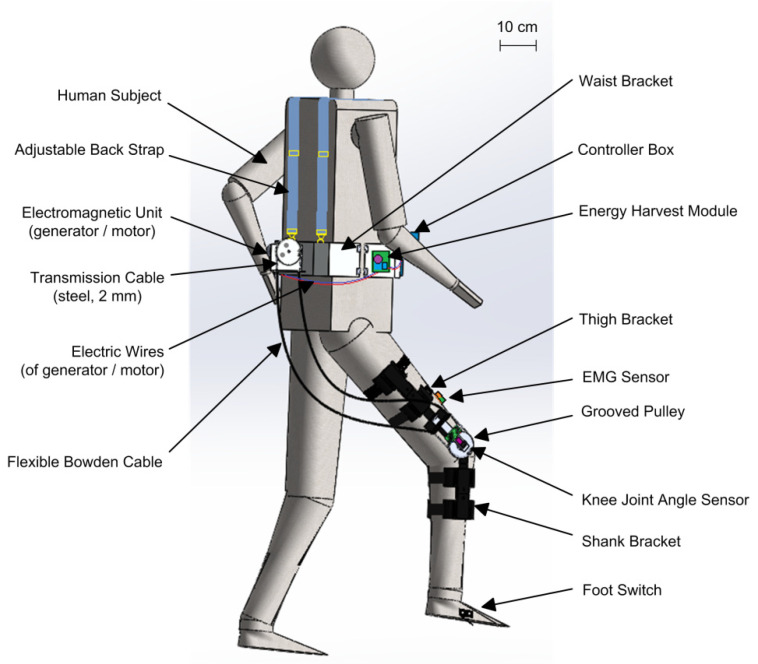
System-level design of the flexible knee joint exoskeleton with the human subject showing. The exoskeleton consists of the waist bracket part, the lower-limb-mounted part (thigh bracket and shank bracket), and the Bowden cable transmission.

The system-level design of the flexible Bowdencable-actuated knee joint exoskeleton robot (with the human subject wearing it) is illustrated in Figure 1 below. The structural model was designed and rendered using Solidworks software (Dassault Systemes SolidWorks Corp., Waltham, MA, USA). To simplify the design, the human subject wears the knee exoskeleton on one of the lower limbs only. Structurally, the exoskeleton consists of three parts: the waist bracket, the lower-limb-mounted part, and the Bowden transmission cable. Other components such as the electromagnetic unit (generator or motor), the energy harvest module, the controller box, and the batteries are mounted on different locations around the waist bracket.

As the human subject walks around, the waist bracket with all these components has a tendency to fall down due to gravity, causing a significant amount of pressure and squeezing on the pelvis of the human subject. To address this issue of wearable discomfort, back straps with adjustable lengths were introduced in our design so that most of the weight of the waist bracket assembly was carried by the shoulder of the human subject instead of the pelvis. Moreover, the adjustable back straps are useful for human subjects with different body sizes when they wear the waist bracket. The lower-limb-mounted part comprises the thigh bracket, the grooved pulley, the knee joint shaft with the angular sensor, and the shank bracket. The knee joint shaft is attached to the thigh bracket, while the grooved pulley is fixed to the shank bracket using bolts. Bearings are installed between the knee joint and grooved pulley to minimize the rotational friction. The transmission cable wraps around the groove of the pulleys, where the cable terminals are embedded or fixed to the outer edge of the pulleys. With proper adjustment of the thigh and shank bracket, the rotational axis of the human subject’s knee joint can be made coaxial with the knee joint shaft and grooved pulley of the exoskeleton so the knee motion of the human subject can be transmitted to the exoskeleton. During human subject movement, the relative distance and angle between the exoskeleton knee brace and the electromagnetic unit (generator or motor) on the waist bracket changes continuously. To reliably transmit the motion between the exoskeleton knee joint and the electromagnetic unit, a flexible Bowden cable transmission system is used to compensate for the hip angle variations during walking. The outer sheath of the Bowden cable is constrained on both ends, and the inner steel cable moves inside the outer sheath, which transmits the motion between the pulleys of the knee and the electromagnetic unit. Here, the radii of the both pulleys are the same, and only the gear box of the electromagnetic unit contributes to the motion amplification between the knee and the generator. Additional sensors, including the EMG sensor and foot switch, are mounted on the thigh and foot, respectively. The major dimensions of the exoskeleton units in Figure 1 are listed in Table 1 below.

During the walking cycle, knee joint flexion and extension occur alternatively and periodically. As the lower limbs accelerate and decelerate, the leg muscles are conducting positive and negative work in the associated gait phases [38,39]. Depending on the specific need, the single electromagnetic unit is designed to function as either an electric motor or a generator. When torque assistance is needed, the electromagnetic unit acts as a motor and outputs mechanical power to the exoskeleton knee joint. When resistance torque or damping is required, the electromagnetic unit switches to the mode of a generator, harvesting the biomechanical energy of the human knee joint. The energy harvest module controls the operational mode switch, which involves data collection and processing of multi-source signals, including the thigh muscle EMG, foot–ground contact switch, and joint angle. Moreover, the harvest module also regulates the degree of energy harvesting such that variable joint damping can be achieved for knee joint impact absorption or for rehabilitation purposes [40,41].

With proper switching of operational modes and careful regulation of energy harvesting, the electromagnetic unit absorbs energy when the muscles consume metabolic energy to perform negative work and outputs torque when the muscles use metabolic energy to perform positive work. As a result, the Bowdencable-actuated exoskeleton with energy harvesting design will reduce muscle activities during the human subject’s walking, and the harvested electric energy, stored in super capacitors or batteries, could be further used to power various electronics, such as a wireless electrocardiographic sensors.

### 2.2. Energy Flow of the Limb-Exoskeleton System and Efficiency

**Figure 2:**
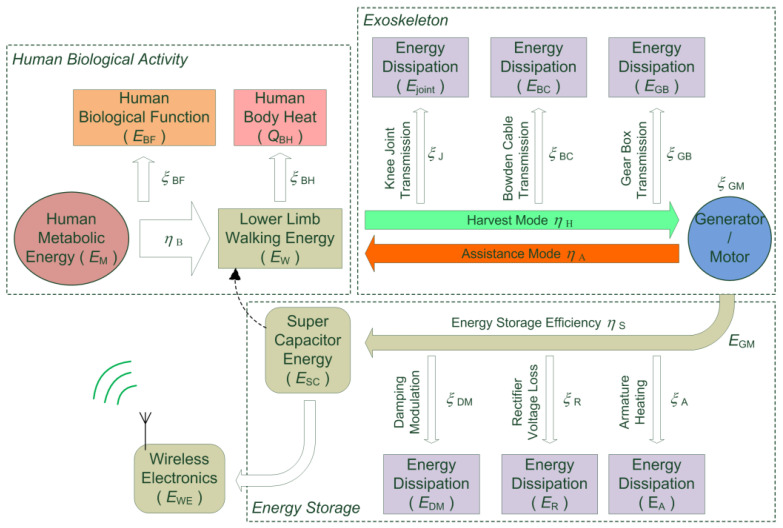
Energy flow and efficiency analysis of a human subject wearing the Bowdencable-actuated knee exoskeleton with an energy harvest module. Here, the symbol *η* represents energy efficiency, and *ξ* represents the coefficient of energy loss from the previous energy block.

As the human subject wears the Bowdencable-actuated knee exoskeleton and walks around, energies of various forms exist in the entire human-exoskeleton system. The associated energy flow is presented in Figure 2 below. Here, all energy terms are represented by the symbol “*E*”, while the transmission efficiency factors are represented by the symbol “*η*” and the coefficient for energy loss is represented by the symbol “*ξ*”. The subscripts for the above three parameters indicate the associated energy flow involved.

Let us assume that the total human metabolic energy generated by the biological activity is *E*_M_, which consists of three major parts: the energy required for maintaining the basic biological functions (*E*_BF_), the dissipated body heat energy (*E*_BH_), and the kinetic energy for lower limb walking (*E*_W_). The associated energy efficiency is denoted as *η*_B_, shown in the human biological activity block in Figure 2.

Under the actions of the lower limb muscles, the knee joint performs extension and flexion alternately. As a result, positive and negative work is generated. The leg swing during the negative work cycle drives the grooved pulley mounted on the knee bracket of the exoskeleton, which pulls the inner cord of the Bowden transmission cable. The transmitted tension of the Bowden cable’s inner cord then drives the grooved pulley attached to the gear box shaft, which further makes the generator rotate at amplified angular speeds. In this process of energy harvesting, however, a certain amount of energy is lost [42]. First, for the knee joint motion transmission, any misalignment between the human subject’s knee axis of rotation and the exoskeleton could lead to a loss of energy (*E*_J_) being transmitted. This is unavoidable due to the movement of clothing between the human subject and the exoskeleton bracket. Next, energy loss (*E*_BC_) caused by backlashes, friction, and nonlinear elasticity exists in the Bowden cable transmission [43,44]. Thus, the rotation of the exoskeleton knee joint could never be fully transmitted to the gear box [45]. Finally, the backlashes and friction in the gearbox contribute to additional energy loss (*E*_GB_). For the positive work cycle, the electromagnetic motor outputs energy to the lower limbs of the human subject, where similar energy loss is involved. Here, *η*_H_ and *η*_A_ represent the energy efficiency of the harvest and assistance modes, respectively.

The electromagnetic generator converts the mechanical energy to electricity, where the generator output energy (*E*_GM_) is produced with an efficiency of *η*_GM_. Typically, the armature resistance, rated as RPM/voltage, and frictions inside the gear reducer of the generator are the crucial specifications for the design. For improved generator efficiency, low armature resistance and gearbox frictions are desired, which reduce the energy losses involved. Moreover, a sufficient output voltage at a relatively lower RPMis required for the generator to output useful power during human movements. The output electricity of the generator, however, needs to be regulated and stored properly [46]. In this paper, the collected electricity is regulated using a Metal-Oxide-Semiconductor Field-Effect Transistor (MOSFET) for knee damping modulation and stored in a super capacitor (*E*_SC_). In this process, an additional amount of electric energy is dissipated, which includes the generator armature heating (*E*_A_), the rectifier diode loss (*E*_R_), and the energy loss in the MOSFET chip (*E*_DM_) during damping modulation. The net energy efficiency for the storage of harvested electricity is denoted as *η*_S_.

From the above analysis, the energy harvesting module of the exoskeleton helps collect a certain amount of energy during the negative work cycle of the human subject’s walking. Therefore, the lower limb walking energy (*E*_W_) is reduced, which subsequently reduces the overall metabolic energy (*E*_M_).

## 3. Theoretical Modeling and Simulation

### 3.1. Dynamic Modeling of Human-Exoskeleton Motion

**Figure 3:**
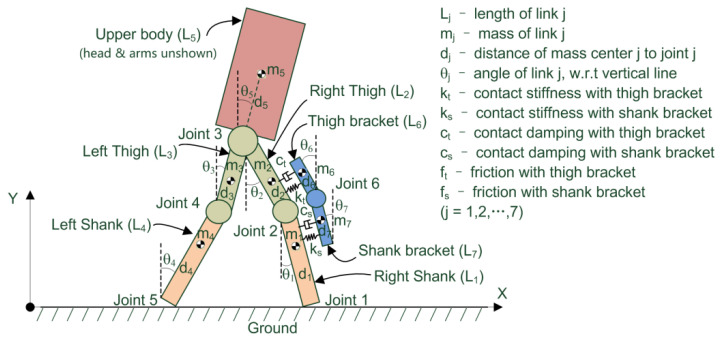
Simplified (5 + 2)-link dynamic model for the human-exoskeleton system, with link parameters and major contact parameters listed and shown in the sagittal plane. Note: the double support phase is presented here, where both feet have ground contact. For the single-support phase, only one foot has ground contact, while the other foot swings in the air.

The dynamic modeling of the human-exoskeleton system is actually rather complicated [47,48]. Strong nonlinearity and time-varying behaviors exist in the complex phenomena [49,50], including foot–ground contact and interactions between the muscles of the human subject, his or her clothing, and the exoskeleton brackets [51,52]. Thus, a complete representation of the dynamic model of the human-exoskeleton system is impossible, nor is it necessary. The theoretical formulations, however, do provide valuable insights into the design, analysis, and optimization of the energy harvester system for the exoskeleton robot. With the help of these theoretical derivations, it is much easier for the designer to focus on the relevant parameters, and some of these theoretical equations could be further converted to empirical equations to assist future designs. In this paper, the human body and exoskeleton are approximated as rigid bodies (namely the links in Figure 3 below), and multi-rigid body dynamics is used to formulate the human-exoskeleton system [53]. As part of the upper portion of the human body (e.g., forearms, upper arms, and hands) does not exhibit as much swinging compared with the lower limbs, the human-exoskeleton model is simplified to a five-link human model with a two-link model for the exoskeleton. The simplified system is created in the sagittal plane, as illustrated in Figure 3.

As seen from Figure 3, the upper body of the human subject (including the head, torso, and arms) is simplified as link 5 (L_5_) as a whole. The right shank and thigh are represented by links 1 (L_1_) and 2 (L_2_), respectively, and the left shank and thigh are labeled as links 4 (L_4_) and 3 (L_3_), respectively. The exoskeleton thigh and shank brackets are represented by links 6 (L_6_) and 7 (L_7_), respectively. To better illustrate each part (i.e., link), the shanks are rendered in carneose colors, with the thighs in yellow, the upper body in purple, and the exoskeleton in blue. The ground underneath is represented by hatched lines. Here, joints 1 and 5 are defined as the foot contact with the ground during the double support phase of walking. Joints 2 and 4 represent the knee joints of both legs of the human body, while joint 6 represents the exoskeleton knee joint. Joint 3 is the hip joint, which connects the lower limbs and the torso of the human subject.

Detailed physical parameters of the simplified human-exoskeleton model are also listed in Figure 3. For the seven simplified links *L*_*j*_ (*j* = 1,2,…7), m_*j*_ represents the mass of link *j* (i.e., each part of the human or exoskeleton), with d_*j*_ being the distance between the mass center and the associated joint, while θ_*j*_ represents the angle between link *j* and the vertical line. The contact interaction between the human’s lower limbs and exoskeleton bracket is modeled by the contact stiffness (k_t_ and k_s_), contact damping (c_t_ and c_s_), and contact friction (f_t_ and f_s_) for the thigh and shank, respectively.

Here, the Lagrangian method is used to formulate the general form of the dynamic equations of the human-exoskeleton system as follows:

(1) $$\frac{d}{dt}\left(\frac{\partial L}{\partial {\dot{q}}_{j}}\right)-\frac{\partial L}{\partial q_{j}}=Q_{j}\;,\;\;j=1,2,\ldots ,N$$

(2) L=K−V

where L is the Lagrange function, which is equal to the difference between the kinetic energy K and the potential energy V of the system, $q_{j}$ is the generalized coordinate which corresponds to the joint angles of the lower limb and exoskeleton bracket, $Q_{j}$ is the generalized force or moment, j is the link number, and N is the total number of degrees of freedom of the human exoskeleton system.

By substituting the model parameters in Figure 3 into Equations (1) and (2), the dynamic equation in state space matrix form can be obtained:

(3) $$M(\theta )\ddot{\theta }+H(\theta ,\dot{\theta })+G(\theta )=T(\theta )$$

where $M\left(\theta \right)$ is the mass and inertia matrix of the human-exoskeleton system, $H\left(\theta ,\dot{\theta }\right)$ is the vector containing the centrifugal and Coriolis terms, $G\left(\theta \right)$ is the vector of gravity terms, and $T\left(\theta \right)$ is the vector of generalized forces or moments applied on the system.

To show the interaction between the human subject and the knee exoskeleton explicitly, the dynamic equation of the entire human-exoskeleton system could be reorganized as Equation (4) for the human subject and Equation (5) for the knee exoskeleton, respectively:

(4) $$M_{H}(\theta_{H}){\ddot{\theta }}_{H}+H_{H}(\theta_{H},{\dot{\theta }}_{H})+G_{H}(\theta_{H})=T_{H}(\theta )+R_{EH}F_{EH}(\theta_{H},\theta_{E},{\dot{\theta }}_{H},{\dot{\theta }}_{E},t)$$

(5) $$M_{E}(\theta_{E}){\ddot{\theta }}_{E}+H_{E}(\theta_{E},{\dot{\theta }}_{E})+G_{E}(\theta_{E})=T_{E}(\theta )+R_{HE}F_{HE}(\theta_{H},\theta_{E},{\dot{\theta }}_{H},{\dot{\theta }}_{E},t)\;$$

where matrices M, H, G, and T follow similar definitions to those in Equation (3), with the subscripts “H” and “E” specifying human and exoskeleton, respectively, R is the coordinate rotational matrix, F is the contact force between the lower limb of the human subject and the exoskeleton bracket, and the subscripts “HE” and “EH” refer to “human to exoskeleton” and “exoskeleton to human”, respectively.

As the human subject is in motion with the knee exoskeleton, a complicated contact phenomenon occurs in between the human skin, clothing, and exoskeleton bracket [54]. Normally, such contact interaction is highly nonlinear and time-varying, which could be rather difficult to model accurately. Obviously, the contact interaction is dependent on the model states of both the human subject and the exoskeleton as well as time, which is indicated on the right-hand side in Equations (4) and (5). Moreover, the contact model is related and sensitive to the clothing materials, human muscle elasticity, knee brace tension, mounting misalignment of the exoskeleton, walking speed and acceleration, operation time, and so on [55].

**Figure 4:**
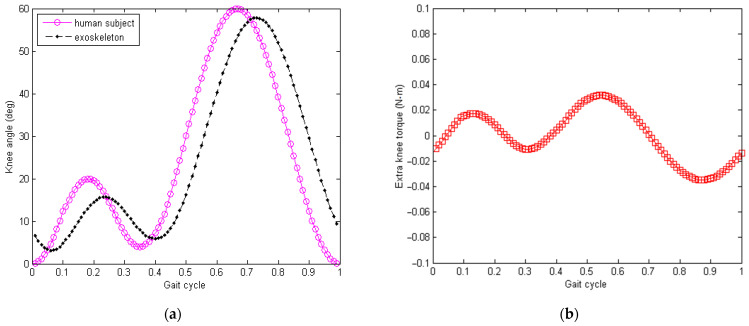
Energy loss due to contact interaction between human subject and exoskeleton bracket. (**a**) Simulated loss of swing amplitude from the knee joint of human subject’s lower limb to the exoskeleton. (**b**) Simulated extra torque load applied to the knee joint of the human subject.

These factors could lead to energy loss from the human’s lower limb to the knee exoskeleton joint. Typical forms of loss include reduced amplitude of the swing for the knee exoskeleton joint and extra torque load to the human subject due to friction, as shown in Figure 4 below. In Figure 4a, the simulated knee joint swing angle is about 56 deg (pk-pk) and 52 deg (pk-pk) for the human subject and exoskeleton, respectively, where 7.14% of the swing amplitude is lost. Figure 4b plots the extra torque applied to the human lower limb. In the simulation, a gait frequency around 120 steps/min was used, which is close to normal human walking [56]. The energy loss in the process of knee motion transmission from the human subject to the exoskeleton contributes to the reduction in the net available power, which could be harvested and generated (i.e., $\xi_{J}$ in Figure 2). This part of energy loss ($\xi_{J}$) could never be fully avoided and will be different from one trial to another, as the contact condition varies every time the knee exoskeleton is mounted on the human subject. Moreover, the energy loss ($\xi_{J}$) also varies with respect to time during the walking process of the human subject wearing the exoskeleton.

### 3.2. Transmission Characterization of the Bowden Cable Actuation System

**Figure 5:**
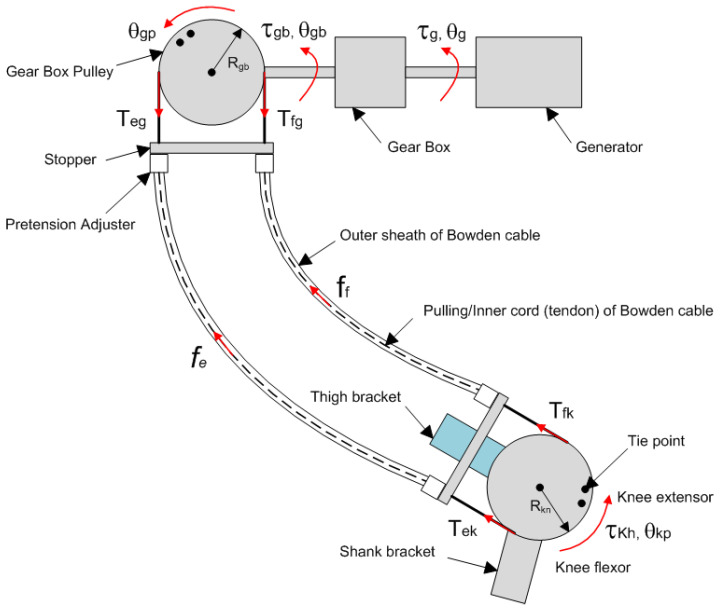
Transmission system of the knee exoskeleton. The Bowdencable actuation system transmits the knee joint motion to the gear box shaft despite the varying relative angle in between. The gear box speeds up the rotation in power generation mode and amplifies the torque in assistance mode.

The knee exoskeleton designed in this paper used a pair of Bowden cables to transmit the rotational motion of the knee joint pulley to the gear box pulley. This would help ensure reliable and continuous motion transmission from the knee joint to the gear box despite the varying relative position and orientation between the knee and gear box during motion. The Bowden cable transmission system, however, does have its limitations [57]. Due to friction between the inner cord (tendon) and outer sheath of the Bowden cable, the input and output tensions are different. Moreover, the input and output displacements of the inner cord (tendon) are different [58]. The schematic of the Bowden cable transmission system is illustrated in Figure 5.

Assuming Coulomb friction inside the outer sheath of the Bowden cable and neglecting the inertia of the inner tendon, the input-output relationship of the inner cord’s tension can be described by the following equations [59]:

(6) $$T_{out}=T_{in}\exp\left(-\mu \mathrm{sgn}\left(\dot{s}\right)\phi \left(L\right)\right)$$

(7) $$\phi \left(L\right)=\int_{0}^{L}\kappa \left(\lambda \right)d\lambda \;$$

where $T_{in}$ and $T_{out}$ are the tensions of the inner cord of the Bowden cable at the input and output ends, respectively, μ is the frictional coefficient between the inner cord and the outer sheath, $\mathrm{sgn}\left(\dot{s}\right)$ gives the pulling direction of the inner cord, L is the length of the tendon, $\kappa \left(\lambda \right)$ is the curvature, and $\phi \left(L\right)$ is the total bending angle of the Bowden cable.

Additionally, the input-output relationship of the tendon displacement can be modeled as

(8) $$S_{out}=S_{in}+\delta \left(s\right)$$

(9) $$\delta \left(s\right)=\left(\int_{0}^{s}\frac{T\left(\lambda \right)}{EA}d\lambda \right)\;$$

where $S_{in}$ and $S_{out}$ are the displacements of the input and output ends of the inner cord, respectively,$\;\delta \left(s\right)$ is the elongation of the inner cord, E is the Young’s modulus of the tendon; and A is its cross-sectional area.

**Figure 6:**
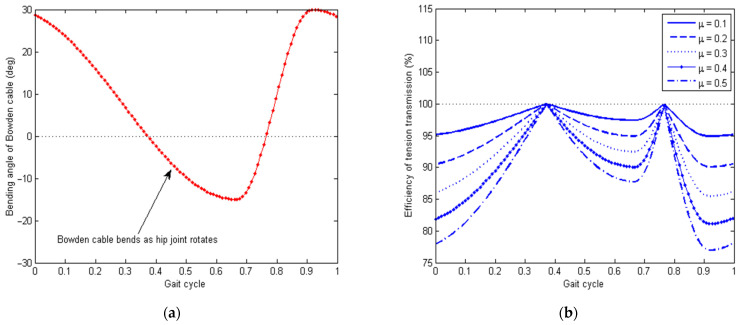
Transmission loss due to Coulomb friction between the inner tendon and outer sheath of the Bowden cable. (**a**) Time history of the bending angle of the Bowden cable caused by the hip joint motion during walking. (**b**) Simulated efficiency of tension transmission of the Bowden cable system.

As can be seen from Equations (6)–(9), the output tension is reduced compared with the input tension due to the friction forces and bending angles of the Bowden cables, and the transmitted displacement is also reduced due to elongation of the inner cord of the Bowden cable. The reduction in both the tendon tension and displacement leads to a loss of energy (i.e., the product of tendon tension and displacement) being transmitted through the Bowden cable system. The energy efficiency of the Bowden cable system is illustrated in Figure 6 below. Here, a typical hip joint signal is used to simulate the time-varying relative angle between the thigh and upper body during walking, as shown in Figure 6a. For different values of the friction coefficient μ, the associated efficiency curves for tension transmission are plotted in Figure 6b. With the increase in the friction coefficient, the transmission efficiency decreased. Hence, for improved transmission efficiency, a special layer of low-friction material (e.g., teflon) and lubrication is needed to reduce the friction coefficient between the inner tendon and outer sheath.

### 3.3. Power Generation by the Knee Exoskeleton

The knee exoskeleton proposed in this paper could operate in both power assistance mode and energy harvesting mode. In power assistance mode, the electromagnetic unit operates as an electric motor. It outputs torque to the gear box, which amplifies the output torque to pull the inner cord of the Bowden cable. The tension further drives the exoskeleton knee joint, which provides torque assistance to the lower limb of the human subject. In energy harvesting mode, the kinetic energy of the human subject’s lower limb drives the exoskeleton knee joint, which ultimately drives the generator through the transmission chain in the opposite manner. Since the focus of this paper is the design and testing of biomechanical energy harvesting, the analysis here mainly deals with the electricity generation process shown in Figure 5 above.

Force balance on the gear box pulley and knee pulley requires the following:

(10) $$\left(T_{eg}-T_{fg}\right)R_{gp}=J_{gp}{\ddot{\theta }}_{gp}+\tau_{gb}$$

(11) $$\left(T_{fk}-T_{ek}\right)R_{kp}=J_{kp}{\ddot{\theta }}_{kp}-\tau_{kh}\;$$

where $T_{eg}$ and $T_{fg}$ represent the inner cord tension applied to the gear box pulley for the knee extensor and flexor, respectively,$\;T_{ek}$ and $T_{fk}$ represent the inner cord tension applied to the exoskeleton knee pulley, $J_{gp}$ and $J_{kp}$ are the moments of inertia for the pulleys attached to gear box and exoskeleton knee joint, respectively,$\;R_{gp}$ and $R_{kp}$ are the associated pulley radii,$\;\theta_{gp}$ and $\theta_{kp}$ are associated angular displacements, and $\tau_{gb}$ and $\tau_{kh}$ are the torque of the gear box shaft and human knee joint, respectively.

By applying Equation (6), the relationship between the differential tensions on the gear box pulley and knee pulley can be written as

(12) $$\left(T_{fg}-T_{eg}\right)=\left(T_{fk}-T_{ek}\right)\exp\left(-\mu \phi \left(L\right)\right)$$

Additional transmission loss exists inside the gear box, which leads to Equation (13):

(13) $$\eta_{gb}\left(\left(T_{eg}-T_{fg}\right)R_{gp}-J_{gp}{\ddot{\theta }}_{gp}\right){\dot{\theta }}_{gp}=\tau_{g}{\dot{\theta }}_{g}$$

where $\eta_{gb}$ is the transmission efficiency of gear box and $\tau_{g}$ and $\theta_{g}$ are the torque and rotation angle of the output shaft of the gear box (or the input shaft of the generator), respectively.

By combining Equations (10)–(13), the transmitted torque on the generator can be obtained as follows:

(14) $$\tau_{g}=\eta_{gb}\left(\left(\tau_{kh}-J_{kp}{\ddot{\theta }}_{kp}\right)\exp\left(-\mu \phi \left(L\right)\right)-J_{gp}{\ddot{\theta }}_{gp}\right)\left(R_{gp}{\dot{\theta }}_{gp}\right)/\left(R_{kp}{\dot{\theta }}_{g}\right)\;$$

Equation (14) establishes the relationship between the original input torque $\tau_{kh}$ from the human subject to the final output torque $\tau_{g}$ applied on the generator.

Moreover, the resistive torque of the generator can also be modeled as

(15) $$\tau_{g}=J_{g}{\ddot{\theta }}_{g}+c_{g}{\dot{\theta }}_{g}\;$$

where $J_{g}$ and $c_{g}$ are the moment of inertia and equivalent damping coefficient of the generator, respectively.

The electric voltage $E_{g}$ produced by the generator during walking can be calculated as follows:

(16) $$E_{g}=K_{g}{\dot{\theta }}_{g}\;$$

where $K_{g}$ is the back electromotive force constant of the generator.

The total electric power, however, depends on the magnitude of the electric current going through the armature coil of the generator. When the generator connects to an open circuit, no electric power is generated in theory due to the zero armature current. Normally, the output terminals of the generator could connect to a load resistor, which disspiates the generated power in the form of heat. In this paper, the generator connects to a super capacitor, which stores the generated electric energy. The calculated total electric power $P_{gR}$ and $P_{gC}$ are described by Equations (17) and (18) below for the resistor load and capacitor load, respectively:

(17) $$P_{gR}=E_{g}^{2}/\left(R_{a}+R_{L}\right)=K_{g}^{2}{\dot{\theta }}_{g}^{2}/\left(R_{a}+R_{L}\right)\;$$

(18) $$P_{gC}=E_{g}\left(E_{g}-U_{SC}\right)/R_{a}=K_{g}{\dot{\theta }}_{g}\left(K_{g}{\dot{\theta }}_{g}-U_{SC}\right)/R_{a}\;$$

(19) $$U_{SC}=\left(\int_{0}^{t}i\left(\tau \right)d\tau \right)/C_{SC}\;$$

where $R_{a}$ is the internal resistance of the generator amarture coil, $R_{L}$ is the load resistance, $U_{SC}$ is the instantaneous voltage of the super capacitor, $C_{SC}$ is the capacitance of the super capacitor, and $i\left(\tau \right)$ is the charging current for the super capacitor.

Since a certain amount of the generated electric power is wasted on the resistive heating of the generator armature, the final output electric power $P_{gR-out}$ and $P_{gC-out}$ are

(20) $$P_{gR-out}={\left(E_{g}/\left(R_{a}+R_{L}\right)\right)}^{2}R_{L}=K_{g}^{2}{\dot{\theta }}_{g}^{2}R_{L}/{\left(R_{a}+R_{L}\right)}^{2}$$

(21) $$P_{gC-out}=U_{SC}\left(E_{g}-U_{SC}\right)/R_{a}=U_{SC}\left(K_{g}{\dot{\theta }}_{g}-U_{SC}\right)/R_{a}$$

**Figure 7:**
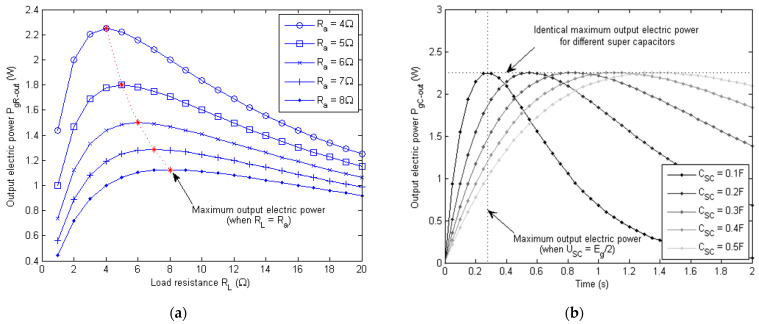
Simulated output electric power of the exoskeleton generator (when $E_{g}=6\;V$) for resistance load and capacitance load. (**a**) Output electric power for generators with different internal resistance and load resistors. (**b**) Time history of the output electric power (when $R_{a}=4\;\Omega$) for different values of capacitance of the super capacitor.

Equations (20) and (21) can be used as empirical equations for the design and optimization of the energy storage system of the generator. The simulated result in Figure 7 indicates that the output power of a given generator is a function of the angular velocity of the exoskeleton knee joint and the external load, which could be a resistor or capacitor. Figure 7a shows that the maximum output power for the resistance load was produced when the load resistance $R_{L}$ equaled or matched the internal resistance of the generator armature $R_{a}$. Moreover, the output power stayed constant with respect to time as long as the generator voltage $E_{g}$ stayed unchanged. The results in Figure 7b show that the maximum output power stayed the same despite different super capacitors being used. However, the maximum output power only happened when the voltage of the super capacitor $U_{SC}$ reached $E_{g}/2$. At the beginning of the charging process, the output power was low, even though the charging current was high, due to the very low voltage of the super capacitor. In the later process of charging, the output power also went down, even though the voltage of the super capacitor was high, due to the reduced charging current.

### 3.4. Impact Absorption Using Controlled Damping

During walking, the angular velocity of the human subject’s knee joint is transmitted to the generator through the exoskeleton bracket, the Bowden cables, and the gear box. The back electromotive force causes an induced current inside the generator armature coil, generating a resistive generator torque $\tau_{gDamping}$ against the input torque $\tau_{g}$. This resistive torque could act as a knee impact absorber and damping [60] when the lower limb of the human subject is performing negative work against gravity (e.g., going downstairs or downhill) or during deceleration (e.g., the leg swing before a heel strike). The damping torque due to the resistor and capacitor loads can be written as

(22) $$\tau_{gRDamping}=c_{gRDamping}{\dot{\theta }}_{g}=\eta_{trans}j_{bc}j_{gb}c_{gRDamping}{\dot{\theta }}_{k}\;$$

(23) $$\tau_{gCDamping}=c_{gCDamping}{\dot{\theta }}_{g}=\eta_{trans}j_{bc}j_{gb}c_{gCDamping}{\dot{\theta }}_{k}\;$$

where $j_{bc}$ and $j_{gb}$ are the transmission ratio of the Bowden cables and gear box, respectively, $\eta_{trans}$ is the overall efficiency for displacement transmission, including all transmission chains, and $\dot{\theta_{g}}$ and ${\dot{\theta }}_{k}$ are the angular velocities of the generator and knee joint, respectively.

The associated damping coefficients $c_{gRDamping}$ (for the resistor load) and $c_{gCDamping}$ (for the capacitor load) are

(24) $$c_{gRDamping}=P_{gR}/{\dot{\theta }}_{g}^{2}=K_{g}^{2}/\left(R_{a}+R_{L}\right)\;$$

(25) $$c_{gCDamping}=P_{gC}/{\dot{\theta }}_{g}^{2}=\left(K_{g}^{2}-K_{g}\left(U_{SC}/{\dot{\theta }}_{g}\right)\right)/R_{a}\;$$

From Equations (24) and (25), it can be seen that the damping coefficient $c_{gRDamping}$ for the resistor load is a constant once the generator model and load resistor are determined. The damping coefficient $c_{gCDamping}$ for the capacitor load, however, varies with the voltage level of the super capacitor $U_{SC}$, as well as the angular velocity of the knee joint ${\dot{\theta }}_{k}$. Equations (24) and (25) are useful for the design and analysis of knee impact absorption.

**Figure 8:**
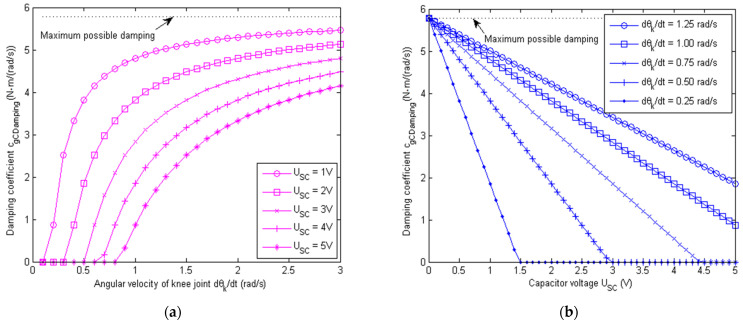
Modulated damping of the exoskeleton generator for capacitance load by controlling $U_{SC}$. Here, the generator armature resistance $R_{a}=6\;\Omega$, back EMF $K_{g}=0.0442\;V/\left(\mathrm{rad}/s\right)$, and total transmission ratio $j_{bc}j_{gb}$ = 133. (**a**) Simulated damping coefficient $c_{gCDamping}$ with the capacitor load as the angular velocity of knee joint ${\dot{\theta }}_{k}$ changes for different super capacitor voltages $U_{SC}$. (**b**) Simulated damping coefficient $c_{gCDamping}$ with capacitor load as the super capacitor voltage $U_{SC}$ changes for different angular velocities ${\dot{\theta }}_{k}$ of the knee joint.

During the operation of the exoskeleton, it is important to modulate the damping of the generator in a controlled manner for terrain-based impact absorption and need-based rehabilitation. The load resistor itself is unable to store any harvested energy, and its resistance value normally could not be adjusted in an automatic manner. The super capacitor, on the other hand, could store the harvested energy for powering other components (e.g., the electrocardiographic sensor), and its voltage level could be controlled in a more flexible manner. The detailed effect of the super capacitor voltage $U_{SC}$ on the damping is illustrated in Figure 8 below.

From Figure 8, it can be seen that the damping coefficient $c_{gCDamping}$ with the capacitor load increased with the angular velocity of the knee joint ${\dot{\theta }}_{k}$. Moreover, it decreased as the charged voltage $U_{SC}$ of the super capacitor rose. Therefore, to maintain sufficient damping for the knee joint, the voltage of the super capacitor should not be too high. In this paper, a modulated damping scheme is proposed by controlling the on and off (i.e., duty cycle) nature of the MOSFET switch and load switch, which regulate the flow of the electric current $i\left(\tau \right)$ into the super capacitor and discharging current to the sensor electronics, respectively. Based on the relationship in Equation (18), the voltage level $U_{SC}$ of the super capacitor could be then regulated, which subsequently controls the damping coefficient $c_{gCDamping}$ as needed.

## 4. Circuit Design for Energy Harvesting

### 4.1. Mode Switching Criteria

**Figure 9:**
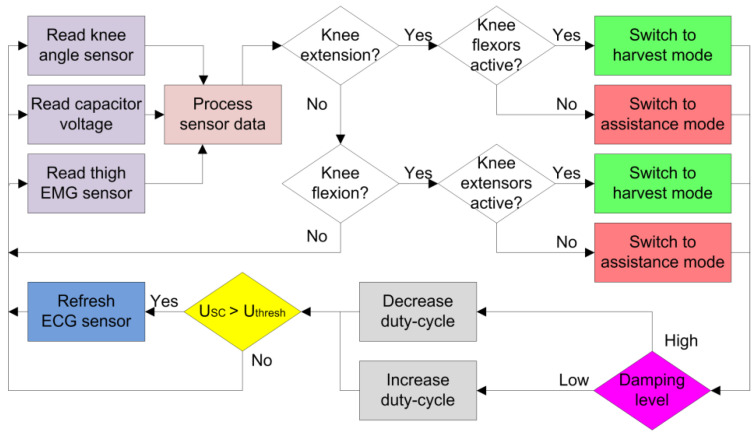
Flow chart of the Bowden cable-actuated knee exoskeleton with dual operational modes for torque assistance or energy harvesting.

For the knee exoskeleton to operate in the dual modes of energy harvesting or torque assistance, the proper mode switching criteria need to be defined. Figure 9 below shows the flow chart of the system. The microcontroller reads the multi-sensor information, including the angular displacement of the exoskeleton knee joint, the EMG signal of thigh muscle activity, and the voltage level of the super capacitor. The multiple sensor data are then processed (in the pink block) to determined the operation state of the system.

When the reading of the angular position of the exoskeleton knee joint decreased, knee extension was performed by the human subject. If the EMG thigh muscle signals indicated an active knee flexor, the knee flexor muscles were acting against the motion of knee extension, and the lower limb of the human subject was performing negative work. In this case, the control system switched to the energy harvesting mode (green blocks) to collect the kinetic or potential energy of the lower limbs. If the EMG thigh muscle signals indicated an active knee extensor, the knee extensor muscles were acting for the motion of knee extension, and the lower limb of the human subject was performing positive work. In this case, the control system switched to the torque assistance mode (red blocks) to help the movements of the lower limbs. Similar mode-switching criteria were implemented for the knee flexion motion.

During energy harvest mode, the electric current produced by the generator is further regulated based on the damping behavior (purple block) of the generator. If there is too much damping, the microcontroller decreases the duty cycle of the power output; otherwise, the microcontroller increases the duty cycle to raise the damping level (purple blocks). The specific damping level could be related to the terrain where the human subject is walking while wearing the exoskeleton. For instance, when going down stairs, the human subject might need more damping for the knee extension process as impact absorption compared with level-ground walking. When the voltage of the super capacitor exceeds some threshold value (yellow block), such as the wake-up voltage of the wireless electrocardiographic (ECG) sensor, the system refreshes the ECG sensor and discharges the electric energy stored in the super capacitor. The system loops and operates as the human participant walks continuously.

### 4.2. Control Circuit with Power Assistance, Energy Storage, and Discharge

**Figure 10:**
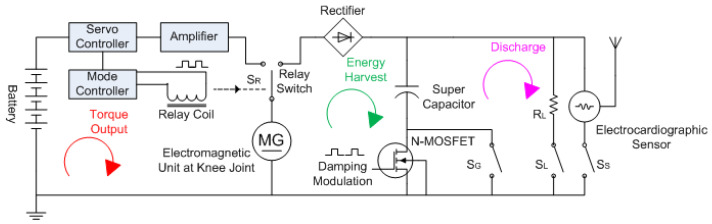
Electric circuit schematic with both the torque output cycle, energy harvesting cycle, and discharge cycle for the Bowden cable-actuated knee exoskeleton.

The control circuit schematic for the knee exoskeleton with dual mode operation is shown in Figure 10 below. Based on the multi-sensor data (including the knee angle and thigh muscle EMG), the operational mode controller sends the mode-switching signal through the relay coil, which controls the relay switch for either the power or torque assistance mode or energy harvesting mode.

During the torque assistance mode, the relay switch $S_{R}$ closes the torque output loop, and the battery energy flows out. The servo controller sends motor control signals to the amplifier and drives the electromagnetic unit (as an electric motor) to output torque at the knee joint for the desired gait motions. The torque assistance mode is normally activated when the muscles of the lower limbs of the human subject are performing positive work.

During energy harvesting mode, the relay switch $S_{R}$ closes the energy harvest loop, and electricity is produced by the electromagnetic unit (as an electric generator). In this mode, the external torque or power is fed in at the knee joint from the human subject. The rectifier of the energy harvest loop ensures the unidirectional flow of electric current into the super capacitor, and the N-channel MOSFET modulates the duty cycle of the charging current such that the charging process and damping can be controlled in a regulated manner.

The discharge loop is activated by the load switches $S_{L}$ and $S_{S}$ for the load resistor $R_{L}$ and the electrocardiographic sensor, respectively. When the super capacitor voltage reaches the turn-on value of the electrocardiographic sensor (@ 3.3 V), the controller closes $S_{S}$ to power on the electrocradiographic sensor. When the voltage of the super capacitor is too high, the load resistor switch $S_{L}$ can be closed to discharge the super capacitor such that the energy harvest loop can continue for the desired knee damping.

**Figure 11:**
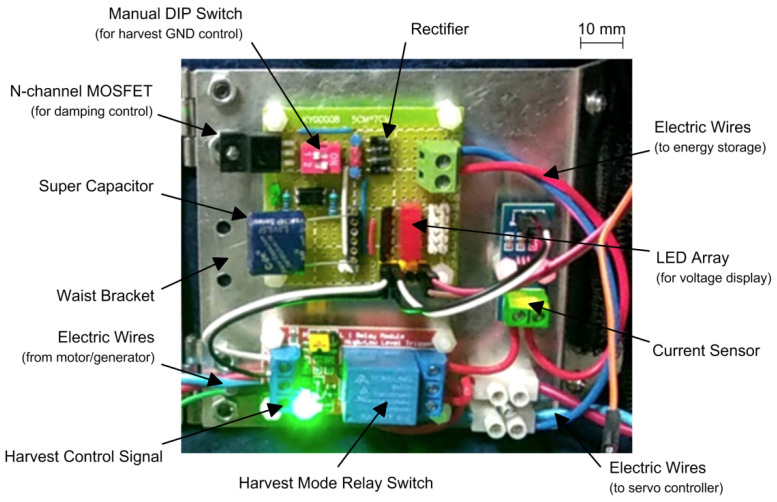
Hardware set-up of the energy harvesting and storage module with operation mode control for the Bowdencable-actuated knee exoskeleton.

The associated hardware circuit board set-up for the energy harvesting and storage module is shown in Figure 11 above. The module consists of the power regulation and control unit (top left), the current sensing module (middle right), the relay switch control block (lower left), and the terminal block (lower right). For the convenience of wiring and demonstration purposes, the energy harvesting module was mounted on the waist bracket. A graphic scale is presented in the top right corner of Figure 11 for the reader to quickly estimate the size of each component. The detailed geometric dimensions and models of the key components in the energy harvesting module are listed in Table 2 below.

## 5. Experimental Validation

### 5.1. Experimental Set-Up

**Figure 12:**
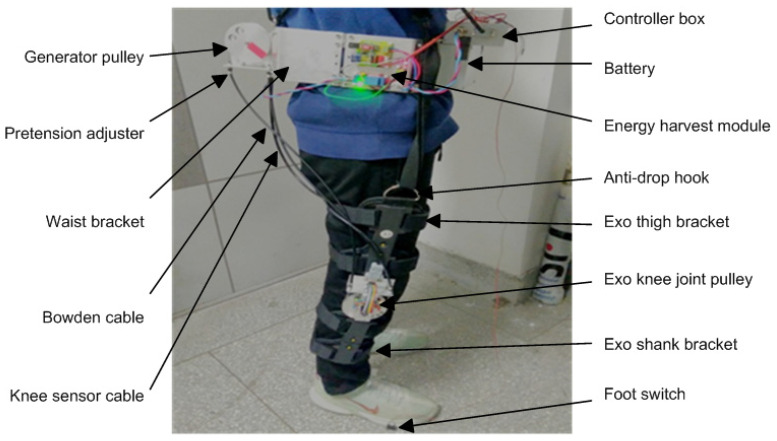
Side view of the prototype of the Bowdencable-actuated knee exoskeleton and the human subject (in a standing position).

The experimental prototype of the Bowdencable-actuated knee exoskeleton with the energy harvesting module is shown in Figure 12 below. The prototype device was built based on the proposed design in Figure 1 in Section 2.1. To reduce the pressure and squeezing on the pelvis part of the human subject, the weight of the waist bracket was supported by the adjustable back straps over the shoulder. Moreover, lightweight materials were used for the components of the knee exoskeleton, including ABS, aluminum, and resin epoxy. The exoskeleton knee brackets were mounted to the thigh and shank of the human body using a Velcro brace so that the knee motion of the human subject could be transferred to the exoskeleton in a compliant manner.

The electromagnetic unit used in this study was the XD-42GA775 generator and motor, which has a back EMF constant $K_{g}=0.0442\;V/\left(\mathrm{rad}/s\right)$, internal armature resistance $R_{a}=6\;\Omega$, and rated rotational speed of 150 rpm. The total transmission ratio of the Bowden cable-actuated system with the generator gear box was $j_{bc}j_{gb}$ = 133. The details of the major parameters of the prototype Bowdencable-actuated knee exoskeleton are listed in Table 3 below.

### 5.2. Experimental Tests and Data Measurements

**Figure 13:**
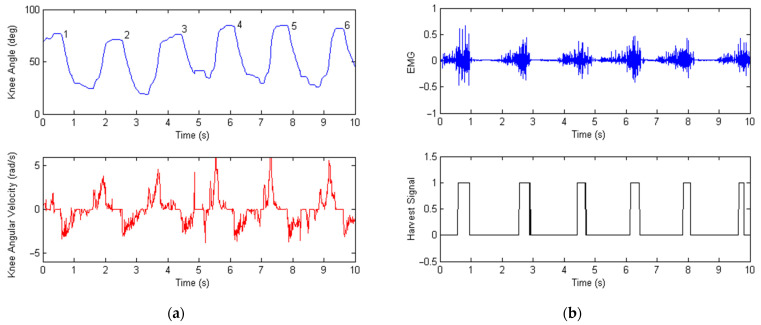
Plots of the measured exoskeleton knee angle and EMG thigh muscle activity with energy harvesting control signals when the walking speed of the human subject was 1.2 km/h downstairs. (**a**) Measured knee angular displacement and velocity of the Bowdencable-actuated knee exoskeleton during movement down stairs. (**b**) Measured EMG signal of the thigh muscle activity and the controlled energy harvest signal.

**Figure 14:**
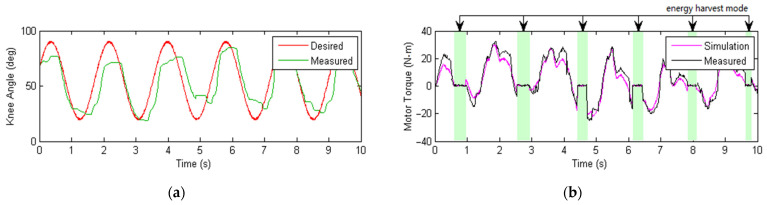
Plots of the measured tracking performance of the exoskeleton knee angle when the walking speed of the human subject was 1.2 km/h downstairs. (**a**) Measured and desired knee angular displacement during movement down stairs. (**b**) Measured motor output or assistance torque showing the mode switching operation of the exoskeleton system. The shaded regions in a light green color correspond to the time intervals of the energy harvest mode, and the remaining regions are for power assistance mode.

**Figure 15:**
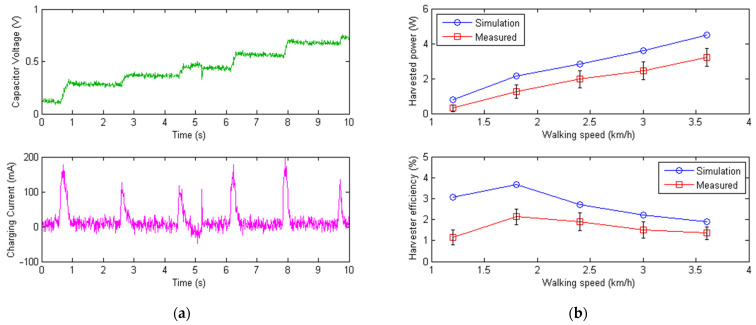
Plots of the measured super capacitor voltage, charging current, and harvest power with efficiency. (**a**) Time history of the measured 0.5F super capacitor voltage and the associated charging current during energy harvesting. (**b**) Simulated and measured harvested power and efficiency (with maximum and minimum shown) at different walking speeds downstairs. As the walking speed increased, the harvested power also increased.

Six healthy human subjects participated in the experimental tests (age: 23 ± 1.6 years; body mass: 61 ± 3.7 kg). All participants provided informed consent, and the test procedures were approved by the Southeast University Research Ethics Board (SEUREB). During the experimental tests, an AD7606 data acquisition module was used to record the voltage signals from various sensors. The acquisition module had a16-bit resolution for analog-to-digital (A/D) conversion. A CJMCU-103 rotary potentiometer was used to measure the knee angle position, and an ACS712-05B current sensor was used to measure the generator current. The data acquisition card was configured to sample at 100 Hz for logging the knee angular position, super capacitor voltage, and generator current. The sampling rate for the EMG muscle activity was 1 kHz. Details of the experimental data are shown in Figure 13, Figure 14 and Figure 15 below.

The time history of the measured knee angular displacement and velocity of the Bowdencable-actuated robot is shown in Figure 13a as the human subject wore the exoskeleton and walked downstairs. The range of the knee swing angle was around 55°, and the walking speed of the human was around 1.2 km/h (0.5-Hz gait frequency). Due to the nonlinear and time-varying contact between the skin, clothing, and exoskeleton knee bracket, the angular displacement had a certain amount of drift. Moreover, the amplitude of each gait cycle could be different, as the human subject could never walk with a perfect gait each time. Figure 13b plots the measured EMG thigh muscle activity and the associated energy harvesting control signal. When negative work was detected (based on the mode switching criteria in Figure 9), the energy harvesting signal became one, collecting the kinetic and potential energy of the human subject. During the energy harvesting process, the generated electricity inside the armature coil of the generator also created a damping or resistive torque, which helped reduce the muscle activity of the human’s lower limb. When positive work was detected, the energy harvesting control signal became zero, and the exoskeleton switched to power assisting mode.

In the experimental test, a simple sinusoidal signal was used as the reference knee angle trajectory with an elementary PI servo control for the power assisting cycle for evaluation purposes. Figure 14a below shows the relationship between the desired knee angle (in red) and the measured knee angle (in green). It can be seen that there was a certain amount of discrepancy between the desired knee angle and the measured one, which could be caused by possible backlashes in the Bowden cable transmission system, the gearbox reducer, or complex nonlinear contact interaction between the human participant and the exoskeleton brackets. Moreover, the imperfect trajectory tracking could also be related to the servo controller’s design and parameter tuning, although this was not the focus of the energy harvesting research in this paper. Despite the tracking error, the proposed exoskeleton offered safe and soft interaction with the human participant without the risk of instability or injuries. Figure 14b shows the output motor torque during operation, where the purple curve is the simulated motor torque and the black curve is the measured one. The shaded regions in light green correspond to the time intervals when the energy harvesting mode was engaged. As can be seen from Figure 14b, the output motor torque was a non-zero value during the torque assistance cycle and around zero during the energy harvesting cycle. Note, however, that the motor still produced damping torque during the energy harvesting mode even though there was no electric power input, which is further analyzed in following parts.

Figure 15a plots the time history of the super capacitor voltage and generator current during energy harvesting. When the human subject performed knee extension, the thigh muscles were performing negative work against gravity, which also brought an impact on the knee joint of the human lower limb. The control system of the exoskeleton switched to energy harvesting mode, and the generator output the electric current. As can be seen from Figure 15, the current peak value was around 200 mA when the human subject was walking at 1.2 km/h downstairs, and the super capacitor voltage rose only when the energy harvesting module engaged. The harvested power and efficiency are shown in Figure 15b at different walking speeds of the human subject going downstairs. As expected, the faster the walking speeds, the higher the harvested power. Here, a maximum harvested power of 3.2 W was obtained at a walking speed of 3.6 km/h, which was close to the normal speed. The harvester efficiency, however, did not simply increase with the walking speed as the harvested power did. When the walking speed was too slow, stictions and discontinuous movements in the transmission system were dominant, leading to low harvester efficiency. When the walking speed was too high, more velocity and torque losses occurred in the transmission process of the knee joint movement from the human body to the exoskeleton. As a result, the efficiency of the harvester reached some optimal point when the walking speed was around a certain value (around 2 km/h) in the experiment. Note that higher walking speeds downstairs were not tested, as they were dangerous and may have caused instability and injuries to the human participants during the test. The summarized data of the harvested power and efficiency are listed in Table 4 below for both the simulated and measured results at different walking speeds.

### 5.3. Analysis of Knee Torque Damping and Muscle Activity

**Figure 16:**
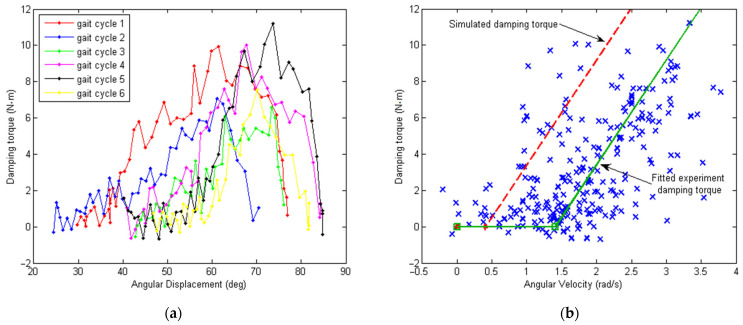
Plots of estimated damping torque based on measured generator current. Only the gait phases with energy harvesting are shown. (**a**) Experimental damping torque $\tau_{gCDamping}$ (with capacitor load) as the angular displacement of the knee joint $\theta_{k}$ changes. The six gait cycles correspond to those in Figure 1. (**b**) Experimental damping torque $\tau_{gCDamping}$ (with capacitor load) as the angular velocity of the knee joint ${\dot{\theta }}_{k}$ changes.

The damping torque produced by the generator in this paper was obtained using the measured current in Figure 15a, where the torque constant $K_{t}=9.55\;K_{g}=0.4221\;\mathrm{Nm}/A$ was applied, which is shown in Figure 16 below. In Figure 16a, the torque–displacement loop is plotted, where only the gait phase with energy harvesting is shown. For the gait cycles without energy harvesting, the damping torque was around zero (not shown for clarity of the plot). In Figure 16b, the torque–velocity loop is plotted. As the angular velocity data were quite noisy due to numerical differentiation, a scatter plot was used with the fitted curve (in solid green), and the simulated damping torque curve was also plotted as a comparison (in dashed red). From Figure 16b, it can be seen that the damping torque was always zero when the angular velocity was below a certain value. This value corresponded to the point where the output voltage of the generator could overcome the sum of the super capacitor voltage ($U_{SC}=1\;V$ here) and rectifier diode voltage loss ($\Delta U_{dr}=0.7\times 2=1.4\;V$). There was also some amount of offset between the simulated damping torque curve and the fitted experimental damping curve. This offset was mainly caused by friction in the system, which lowered the damping effect of the generator.

**Figure 17:**
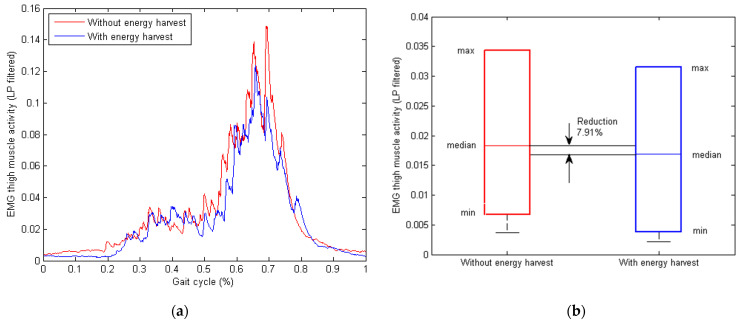
Plot and comparison of the measured EMG thigh muscle activity without and with energy harvesting when the walking speed of the human subject was 1.2 km/h downstairs. (**a**) Comparison of the averaged EMG thigh muscle signal from the experiment in one gait cycle (rectified and low-pass filtered). (**b**) Box plot of the averaged EMG thigh muscle activity from the experiment, where a reduction of 7.91% was observed from the experimental tests.

A comparison of the EMG thigh muscle activity with and without the energy harvesting control is presented in Figure 17. The EMG signals here were rectified and low-pass filtered for better presentation of the thigh muscle activities, as shown in Figure 17a. The box plots of the EMG signals are plotted in Figure 17b, where a reduction of 7.91% for the median EMG signal was observed when there was energy harvesting.

## 6. Conclusions

In this paper, a flexible Bowdencable-actuated lower limb knee exoskeleton was developed with the capability of both assisting human movements and harvesting kinetic energy from the human body. The electromagnetic unit can operate as a generator and impact absorber for the knee joint when the lower limb decelerates or goes downstairs. Unlike conventional mechanical dampers, the effective electrical damping of the generator during the energy harvesting process can be adjusted and regulated by the microcontroller based on the user’s specific needs during operation. The harvested energy is regulated and stored in super capacitors to power wireless electronics when needed. The experimental results showed an average of a 7.91% reduction in thigh muscle activity, with a maximum of 3.2 W of electric power being generated during movement down stairs. As a result, the proposed energy harvester design in this paper offers better wearing synergy when compared with previous designs (e.g., the bulky “backpacks” [21,22]). Furthermore, it combines the advantages of previous devices for both the soft and unpowered exoskeletons for energy harvesting (e.g., [7,24,25]). However, friction in the Bowdencable transmission and gear box as well as the internal resistance of the generator armature caused a significant amount of loss during the energy harvesting process, which will be the focus of improvement in future research. Even so, the proposed design offers important prospects for the realization of lightweight wearable exoskeletons with improved efficiency and long-term sustainability.

## Figures and Tables

**Table 1:** Major dimensions of the flexible Bowdencable-actuated knee exoskeleton robot.

Exoskeleton Unit	Dimension ^1^	Unit
Waist bracket	100 (L) × 10 (W) × 0.1 (H)	cm
Thigh bracket	30 ± 5 (L) × 7.5 (W) × 1.5 (H)	cm
Shank bracket	25 ± 5 (L) × 7.5 (W) × 1.5 (H)	cm
Adjustable back strap	80 ± 20 (L) × 2.5 (W)	cm
Electromagnetic unit	10 (L) × 4.5 (D)	cm
Transmission cable (inner cord)	900 (L) × 2 (D)	mm
Bowden cable (outer sheath)	600 (L) × 5 (D)	mm
Controller box	15 (L) × 10 (W) × 6 (H)	cm
Energy harvest module	9 (L) × 9 (W) × 1.5 (H)	cm
EMG sensor	60 (L) × 30 (W) × 4 (H)	mm
Grooved pulley	1 (H) × 8 (D)	cm
Knee joint angle sensor	30 (L) × 15 (W) × 3 (H)	mm
Foot switch	20 (L) × 10 (W) × 5 (H)	mm

^1^ L: length; H: height; W: width; D: diameter.

**Table 2:** Geometric dimensions of the major components in the energy harvesting module.

Component	Dimensions ^1^ or Model	Unit
Power regulation and control unit	50 (L) × 50 (W) × 25 (H)	mm
Relay switch control unit	50 (L) × 20 (W) × 35 (H)	mm
Current sensing module	30 (L) × 10 (W) × 20 (H)	mm
Terminal block	15 (L) × 15 (W) × 15 (H)	mm
Super capacitor (0.5 F)	CHP5R5L-504R-TW	-
N-MOSFET	FQPF10N60C	-
Rectifier diodes	1N4001	-
Relay switch	JQC-3FF-SZ	-

^1^ L: length; H: height; W: width; D: diameter.

**Table 3:** Major parameters of Bowdencable-actuated knee exoskeleton.

Parameter	Symbol	Value	Unit
Weight of waist bracket assembly (without battery)	*Mwb*	1150	g
Weight of knee bracket assembly	*Mkb*	720	g
Radius of Bowden cable’s inner cord	*Ric*	2	mm
Total length of Bowden cable	*Lbc*	600	mm
Bending angle of Bowden cable	*φbc*	−10~110	deg
Pretension adjustment of Bowden cable	Δ*Lbc*	−6~0	mm
Gearbox backlash of generator	Δ*βgb*	±2	deg
Armature resistance of generator	*Ra*	6.2	Ω
Back EMF (electromotive force) of generator	*Kg*	0.0442	V/(rad/s)
Gear ratio of generator	*jgb*	133	-
Rated speed of generator	*n0*	150	rpm
Materials	*-*	Al, ABS	-
Super capacitor	*Csc*	0.5	F
Sensitivity of current sensor	*λcs*	185	mV/A
Voltage loss of diode rectifier	Δ*Udr*	0.7	V

**Table 4:** Summary of the harvested power and efficiency at different walking speeds.

Walking Speed	Harvested Power Simulated and Measured	Harvester Efficiency Simulated and Measured
1.2 km/h	0.80 W 0.30 W	3.07% 1.15%
1.8 km/h	2.15 W 1.25 W	3.67% 2.13%
2.4 km/h	2.81 W 1.97 W	2.71% 1.89%
3.0 km/h	3.60 W 2.45 W	2.21% 1.51%
3.6 km/h	4.48 W 3.23 W	1.91% 1.38%

## Data Availability

The data used to support the findings of this study are available from the corresponding author upon request.
